# Evaluation of the clinical benefit of an electromagnetic navigation system for CT-guided interventional radiology procedures in the thoraco-abdominal region compared with conventional CT guidance (CTNAV II): study protocol for a randomised controlled trial

**DOI:** 10.1186/s13063-017-2049-6

**Published:** 2017-07-06

**Authors:** RC. Rouchy, A. Moreau-Gaudry, E. Chipon, S. Aubry, L. Pazart, B. Lapuyade, M. Durand, M. Hajjam, S. Pottier, B. Renard, R. Logier, X. Orry, A. Cherifi, E. Quehen, G. Kervio, O. Favelle, F. Patat, E. De Kerviler, C. Hughes, M. Medici, J. Ghelfi, A. Mounier, I. Bricault

**Affiliations:** 1 Clinique Universitaire de Radiologie et Imagerie Médicale, Centre Hospitalier Universitaire (CHU) de Grenoble-Alpes, F-38000 Grenoble, France; 2 Institut national de la santé et de la recherche médicale (Inserm) Centre d’Investigation Clinique (CIC) 1406, University Grenoble-Alpes, F-38000 Grenoble, France; 3 Institut national de la santé et de la recherche médicale (Inserm) Centre d’Investigation Clinique (CIC) 1406, F-38000 Grenoble, France; 4 Pole Recherche, Centre Hospitalier Universitaire (CHU) de Grenoble-Alpes, F-38000 Grenoble, France; 5 Techniques de l’Ingénierie Médicale et de la Complexité – Informatique, Mathématiques et Applications, Grenoble (TIMC-IMAG), University Grenoble-Alpes, F-38000 Grenoble, France; 6 Techniques de l’Ingénierie Médicale et de la Complexité – Informatique, Mathématiques et Applications, Grenoble (TIMC-IMAG), Centre national de la recherche scientifique (CNRS), F-38000 Grenoble, France; 7 Pole Sante Publique, Centre Hospitalier Universitaire (CHU) de Grenoble-Alpes, F-38000 Grenoble, France; 8 Service de Radiologie Ostéo-Articulaire, Centre Hospitalier Universitaire (CHU) Besançon, F-25000 Besançon, France; 9 Institut national de la santé et de la recherche médicale (Inserm) Centre d’Investigation Clinique (CIC) 1431, F-25000 Besançon, France; 10 Service d’Imagerie Diagnostique et Therapeutique, Centre Hospitalier Universitaire (CHU) Bordeaux, F-33000 Bordeaux, France; 11 Institut national de la santé et de la recherche médicale (Inserm) Centre d’Investigation Clinique (CIC) 1401, F-33000 Bordeaux, France; 12 Centre d’Investigation Clinique (CIC) 1401, University Bordeaux, F-33000 Bordeaux, France; 13 Centre Hospitalier Universitaire (CHU) Bordeaux, F-33000 Bordeaux, France; 14 Service de Radiologie, Hôpital Ambroise-Paré, Assistance Publique-Hôpitaux de Paris (AP-HP), F-92100 Boulogne-Billancourt, France; 15 Institut national de la santé et de la recherche médicale (Inserm) Centre d’Investigation Clinique (CIC) 1429, Hôpital Raymond-Poincaré, Assistance Publique-Hôpitaux de Paris (AP-HP), F-92380 Garches, France; 16 Service de Radiologie, Centre Hospitalier Universitaire (CHU) Lille, F-59000 Lille, France; 17 Institut national de la santé et de la recherche médicale (Inserm) Centre d’Investigation Clinique (CIC) 1403, Centre Hospitalier Universitaire (CHU) Lille, University Lille, F-59000 Lille, France; 18 Service de Radiologie, Centre Hospitalier Régional Universitaire (CHRU) de Nancy, F-54000 Nancy, France; 19 Institut national de la santé et de la recherche médicale (Inserm) Centre d’Investigation Clinique – Centre de technologie innovante (CIC-IT) 1433, Centre Hospitalier Régional Universitaire (CHRU) de Nancy, F-54000 Nancy, France; 20 Service Imagerie Abdominale et Générale, Centre Hospitalier Universitaire (CHU) Rennes, F-35000 Rennes, France; 21 Institut national de la santé et de la recherche médicale (Inserm) Centre d’Investigation Clinique (CIC) 1414, Centre Hospitalier Universitaire (CHU) Rennes, F-35000 Rennes, France; 22 Département Imagerie Médicale, Centre Hospitalier Universitaire (CHU) Tours, F-37000 Tours, France; 23 Institut national de la santé et de la recherche médicale (Inserm) Centre d’Investigation Clinique (CIC) 1415, Centre Hospitalier Universitaire (CHU) Tours, F-37000 Tours, France; 24 Service de Radiologie, Hôpital Saint Louis, Assistance Publique-Hôpitaux de Paris (AP-HP), F-75475 Paris, France

**Keywords:** Computed tomography, Computer-assisted medical intervention, Electromagnetic navigation, IMACTIS-CT®, Imaging guidance, Interventional radiology, Medical device, Minimally invasive

## Abstract

**Background:**

Interventional radiology includes a range of minimally invasive image-guided diagnostic and therapeutic procedures that have become routine clinical practice. Each procedure involves a percutaneous needle insertion, often guided using computed tomography (CT) because of its availability and usability. However, procedures remain complicated, in particular when an obstacle must be avoided, meaning that an oblique trajectory is required. Navigation systems track the operator’s instruments, meaning the position and progression of the instruments are visualised in real time on the patient’s images. A novel electromagnetic navigation system for CT-guided interventional procedures (IMACTIS-CT®) has been developed, and a previous clinical trial demonstrated improved needle placement accuracy in navigation-assisted procedures. In the present trial, we are evaluating the clinical benefit of the navigation system during the needle insertion step of CT-guided procedures in the thoraco-abdominal region.

**Methods/design:**

This study is designed as an open, multicentre, prospective, randomised, controlled interventional clinical trial and is structured as a standard two-arm, parallel-design, individually randomised trial. A maximum of 500 patients will be enrolled. In the experimental arm (navigation system), the procedures are carried out using navigation assistance, and in the active comparator arm (CT), the procedures are carried out with conventional CT guidance. The randomisation is stratified by centre and by the expected difficulty of the procedure. The primary outcome of the trial is a combined criterion to assess the safety (number of serious adverse events), efficacy (number of targets reached) and performance (number of control scans acquired) of navigation-assisted, CT-guided procedures as evaluated by a blinded radiologist and confirmed by an expert committee in case of discordance. The secondary outcomes are (1) the duration of the procedure, (2) the satisfaction of the operator and (3) the irradiation dose delivered, with (4) subgroup analysis according to the expected difficulty of the procedure, as well as an evaluation of (5) the usability of the device.

**Discussion:**

This trial addresses the lack of published high-level evidence studies in which navigation-assisted CT-guided interventional procedures are evaluated. This trial is important because it addresses the problems associated with conventional CT guidance and is particularly relevant because the number of interventional radiology procedures carried out in routine clinical practice is increasing.

**Trial registration:**

ClinicalTrials.gov identifier: NCT01896219. Registered on 5 July 2013.

**Electronic supplementary material:**

The online version of this article (doi:10.1186/s13063-017-2049-6) contains supplementary material, which is available to authorized users.

## Background

Interventional radiology is a diagnostic and therapeutic specialty that includes a wide range of minimally invasive image-guided procedures [1, 2]. Therapeutic procedures include intra*-*articular corticosteroid injection, abscess drainage and radiofrequency tumour ablation, whereas diagnostic procedures involve reaching a target to obtain a sample for further analysis. The common denominator of all these procedures is the percutaneous needle insertion step: the guidance of a needle to a defined target.

Different imaging modalities are used to guide the needle through the body to the target, such as radiography, computed tomography (CT), ultrasound (US) and magnetic resonance imaging (MRI) [3–6]. CT guidance provides invaluable assistance [7–9] and has been shown to be more accurate than US-guided procedures [10]. Furthermore, compared with MRI, it is more accessible and has a lower associated cost and a more practical environment (magnetic field compatibility, smaller machine size).

Despite the aid supplied by CT guidance, procedures remain complicated, in particular when an obstacle must be avoided (e.g., the posterior pleural cavity during adrenal gland punctures), meaning that the optimal needle path is on a plane that is oblique to the acquired axial images. An out-of-plane trajectory is associated with decreased needle placement accuracy, leading to trajectory errors which could cause perforation of neighbouring organs or insufficient treatment delivery. Furthermore, radiation exposure increases, particularly in complex procedures performed by unskilled radiologists [11]. This is due mainly to the fact that the radiologist has no means other than fluoroscopy to verify the needle’s progress in real time [12], leading to increased radiation exposure for both the patient and the operator. Therefore, the operator may choose a non-optimal axial trajectory rather than the optimal, difficult oblique trajectory.

Surgical navigation systems involve first the generation of an accurate 3D model of the patient. The operator then uses instruments that are tracked by the navigation system, meaning that the progression of the instruments is shown on the images of the patient, and their position with respect to the patient’s anatomy can be visualised by the operator in real time [13]. Such navigation systems have demonstrated their value in the fields of orthopaedics [14, 15], urology [16] and neurosurgery [17–20], as well as for percutaneous renal punctures [21] and for use by ear, nose and throat specialists [22]. In the field of interventional radiology, navigation can be achieved using CT fluoroscopy. Though the progression of the needle can be monitored in real time, the technique increases the radiation dose delivered [13, 23, 24], and furthermore, specific expertise is required to analyse in real time the acquired CT fluoroscopic images.

**Fig. 1:**
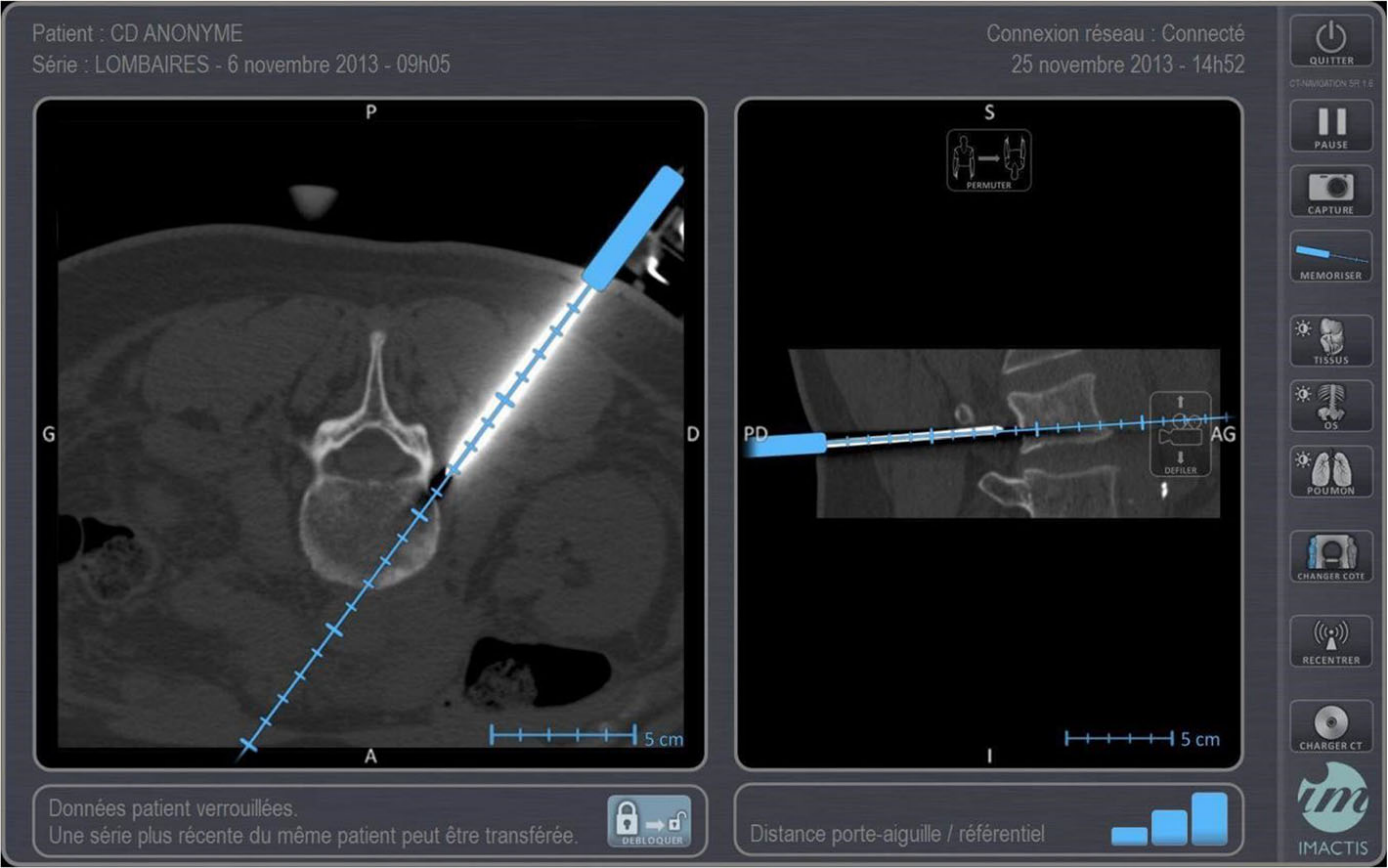
Screenshot of a computed tomography-guided percutaneous biopsy of a spinal lesion. The biopsy has been carried out using navigation assistance provided by the IMACTIS-CT® system. It can be seen that the estimated position of the trocar (*blue*) is accurate with respect to the real position of the trocar (*white*)

The start-up company IMACTIS® (Grenoble, France), in close collaboration with the Techniques de l’Ingénierie Médicale et de la Complexité – Informatique, Mathématiques et Applications, Grenoble (Medical Engineering and Complexity Techniques – Computer Science, Mathematics and Applications, Grenoble; TIMC-IMAG), Grenoble-Alpes University, Grenoble, France, has developed a new electromagnetic navigation system for CT-guided interventional radiology procedures (IMACTIS-CT®). The patient’s anatomy is visualised in 3D using a previously acquired CT scan. An electromagnetic transmitter is attached to the patient’s skin, which enables the needle used by the operator and equipped with an electromagnetic receiver to be located. The hypothetical needle trajectory can therefore be displayed in real time on the images of the patient [25] (*see* Fig. 1). The operator can visualise the trajectory of the needle during both the planning phase (determination of the optimal route before skin penetration) and the needle insertion phase of the procedure.

Previous studies have shown that use of the navigation system for CT-guided interventions results in high needle placement accuracy, even if the target requires an out-of-plane trajectory [26, 27]. The feasibility of oblique trajectories means that the number of possible needle trajectories is increased when using the navigation system compared with conventional CT guidance. The navigation system should therefore increase both the accuracy of the gesture and the radiologist’s confidence in his gesture, whereas the radiation exposure and the duration and severity of the intervention should decrease [28].

The accuracy of the IMACTIS-CT® navigation system has previously been demonstrated by the results obtained in a monocentric, prospective, randomised, controlled clinical trial (ClinicalTrials.gov identifier: NCT00828893). Among 120 patients undergoing routine percutaneous CT procedures enrolled at the Grenoble-Alpes University Hospital, half of the procedures were carried out with the assistance of the navigation system and half were carried out using conventional CT guidance. According to the intention-to-treat (ITT) analysis, the use of the navigation system improved the needle placement accuracy; the median (interquartile range) distance error (in millimetres) using navigation-assisted guidance was 4.1 [2.7–9.1] compared with that using conventional guidance, which was 8.9 [4.9–15.1] (*p* < 0.001). Furthermore, according to the per-protocol (PP) analysis, the use of the navigation system reduced the number of control CT scans acquired during the needle insertion; using navigation-assisted guidance, a median of 2 [2, 3] scans were required, compared with conventional guidance, which required a median of 3 [2–4] scans (*p* = 0.01) [29].

This first clinical evaluation of the system enabled the pertinence of navigation assistance for CT-guided interventional radiology to be confirmed. However, to accurately evaluate the clinical benefit of the device, it is necessary to test the navigation system within a wider medical community. This paper describes an open, multicentre, prospective, randomised, controlled clinical trial that has been designed to evaluate the clinical benefit, in terms of safety, efficacy and performance, of using the IMACTIS-CT® system for navigation-assisted CT guidance compared with conventional CT guidance during interventional radiological procedures in the thoraco-abdominal region.

## Methods/design

### Study design and setting

This study is an open, multicentre, prospective, randomised, controlled interventional clinical trial designed to evaluate the clinical benefit of the IMACTIS-CT® navigation system compared with the conventional method of CT-guided interventional radiological procedures in the thoraco-abdominal region. The nature of the procedures is open, including biopsies, abscess drainage, tumour ablation by radiofrequency or cryotherapy, and intra*-*articular corticosteroid injection procedures.

The trial is sponsored by the Grenoble-Alpes University Hospital (Isère, France). Eight further hospitals are participating in the study: Ambroise-Paré University Hospital (Hauts-de-Seine, France), Besançon University Hospital (Doubs, France), Bordeaux University Hospital (Gironde, France), Lille University Hospital (Nord, France), Nancy University Hospital (Meurthe-et-Moselle, France), Rennes University Hospital (Ille-et-Vilaine, France), Saint-Louis University Hospital (Paris, France) and Tours University Hospital (Indre-et-Loire, France). Also participating in this clinical trial are the eight centres of the Clinical Investigation Centre for Innovative Technology (CIC-IT; www.cic-it.fr) network (Besançon, Bordeaux, Garches, Grenoble, Lille, Nancy, Rennes and Tours), as well as the start-up company IMACTIS® (Grenoble, France; www.imactis.com), which developed the novel electromagnetic navigation system for CT-guided interventional radiological procedures in close collaboration with the TIMC-IMAG (Grenoble-Alpes University, Grenoble, France; www-timc.imag.fr), which specialises in medical engineering, informatics and mathematics.

For this trial, a maximum of 500 patients for whom a percutaneous procedure in the thoraco-abdominal area under CT guidance has been prescribed will be enrolled in the study across the nine participating hospitals. The trial is structured as a standard two-arm, parallel-design, individually randomised trial*.* Patients are randomly assigned to the experimental arm of the trial in which procedures are carried out using navigation assistance (the NAV group), where the procedures are carried out using navigation assistance supplied by the medical device under evaluation (IMACTIS-CT®), or to the active comparator arm of the trial in which procedures are carried out guided by conventional CT (CT group), where the procedures are carried out under conventional CT guidance. The randomisation is stratified by centre and by the expected difficulty of the procedure. The assignment is open label for both the radiologist and patient.

Because a previous clinical trial has already demonstrated improved needle placement accuracy in navigation-assisted procedures using the IMACTIS-CT® device (4.1 mm [2.7–9.1]) compared with conventional procedures (8.9 mm [4.9–15.1], *p* < 0.001) according to ITT analysis [29], the aim of this trial is therefore to evaluate the clinical benefit of the navigation system. The primary outcome is a combined criterion composed of three different criteria which assess, respectively, the safety, efficacy and performance of navigation-assisted CT-guided procedures by comparing the results obtained in the navigation-assisted group with those obtained in the conventional group. The criteria will be evaluated by a blinded expert committee. It will be determined that the navigation system represents a clinical benefit if non-inferiority in terms of safety, efficacy and superiority in terms of performance is obtained in the navigation-assisted group with respect to the conventional group. Also evaluated, in terms of secondary outcomes, are (1) the duration of the procedure, (2) the satisfaction of the operator and (3) the irradiation dose delivered, with (4) subgroup analysis according to the expected difficulty of the procedure, as well as an evaluation of (5) the usability of the device.

The clinical trial is overseen by the trial steering committee (TSC), composed of I. Bricault (coordinating investigator, Grenoble-Alpes University Hospital), A. Moreau-Gaudry (medical coordinator of CIC-IT Grenoble), L. Carrat (president of IMACTIS®), F. Barbot (coordinator of CIC-IT Garches) and Y. Gandon (investigator, Rennes University Hospital). The TSC has responsibility for monitoring the patient enrolment rate at each centre and modifying the proposed statistical analysis framework if necessary following the interim analysis.

### Materials

The medical device under evaluation in this clinical trial (IMACTIS-CT®) is an electromagnetic navigation system for CT-guided interventional radiological procedures. The device was Conformité Européenne (European Conformity; CE)-marked in 2013. Because it is an active medical device designed to be used in the field of diagnostic and therapeutic interventional radiology, it is identified as a class IIA medical device according to the European legal framework. The device is composed of the following components:

1. A navigation station that includes:
  - A computer
  - A touchscreen
  - An electromagnetic transmitter to be attached to the patient’s skin
  - An electromagnetic receiver to be attached to the needle holder
2. Navigation software that has been specifically designed for CT-guided percutaneous procedures
3. A packet of consumables that includes:
  - A single-use sterile needle holder
  - A single-use sterile drape to cover the receiver
  - Hardware that contains the software parameters and which enables the software to be launched

The operating principle of the navigation system is based on the localisation (position and orientation) in real time of the needle holder, equipped with an electromagnetic receiver, with respect to the patient by means of the electromagnetic transmitter that is attached to the patient’s skin. A registration process enables a previously acquired CT scan of the patient to be positioned with respect to the current anatomy of the patient. The operator can then visualise the position of his instrument in real time on the CT images. Therefore, during the planning phase, the hypothetical trajectory of the needle, calculated from the current position of the needle holder, can be visualised to determine the optimal route before skin penetration, and during the needle insertion phase of the procedure, the operator can visualise the progress of the needle through the body in real time.

The president of IMACTIS® and/or an application engineer from the company will install the navigation system at each of the nine participating hospitals and will then provide training for the participating radiologists on how to use the system. Training is obligatory before any radiologist is authorised to carry out a navigated procedure on a patient enrolled in the trial. Training is first conducted using phantom models and is then continued on one or more patients who are not enrolled in the trial. The number of training procedures carried out is noted to minimise the learning curve bias.

For this clinical trial, the foreseeable risks are those associated with conventional CT-guided interventional radiology. No foreseeable supplementary risk is expected from the use of the navigation system. Contraindications are explicitly stated in the inclusion and exclusion criteria. Furthermore, because the navigation system is a passive medical device, the radiologist can choose to stop using the system at any moment, in particular in case of failure or malfunction of the system, and complete the procedure under conventional CT guidance, without any influence on the procedure or patient.

### Methods

For a patient to be considered eligible for study participation, the patient must answer ‘yes’ to all of the following inclusion criteria:

- Aged ≥18 years
- Patients for whom a percutaneous diagnostic or therapeutic interventional procedure in the thoraco-abdominal area under CT guidance has been prescribed and consensually agreed by a multidisciplinary team of radiologists, surgeons and clinicians
- Patients affiliated with the social security (or similar) system
- Patients who have signed the consent certificate of the informed consent form of the trial

Furthermore, to be considered for enrolment, the patient must answer ‘no’ to all of the following exclusion criteria:

- Patients with non-MRI-compatible devices or implanted material (e.g., pacemaker)
- Patients with implanted ferromagnetic material in the thoraco-abdominal area that could interfere with the navigation system
- Pregnant women and lactating mothers
- Persons who are wards of court or under guardianship
- Persons deprived of freedom by judicial or administrative decision
- Persons under legal protection

Patients eligible for enrolment in the clinical trial will be informed of the study during their pre-intervention consultation with a clinical trial investigator, who will deliver clear, intelligible and objective information; answer questions; and verify the inclusion and exclusion criteria. A reflection period is then respected, of durations varying from 2 h to 1 week before the intervention. If the patient chooses to participate in the study, the consent certificate is signed, and the patient is enrolled in the trial. To minimise the withdrawal of consent, the consent certificate is signed as closely as possible to the moment at which the procedure begins.

An initial CT scan of the patient is acquired before assignment of the patient into the experimental or comparator arm of the trial. First, landmarks are made on the patient’s skin using metal wire or a metal grill (required for conventional CT guidance), and the electromagnetic transmitter of the navigation system is attached to the patient’s skin (required for navigation-assisted CT guidance). The CT scan is then acquired, and it is verified that the procedure originally prescribed is maintained and that the navigation system is available and working. If the originally prescribed procedure is no longer maintained or the navigation system is not available or not working, the patient is removed from the study, and the procedure is carried out under conventional CT guidance.

Otherwise, the radiologist estimates the expected difficulty of the procedure on the basis of the initial CT scan. A procedure is expected to be difficult if the needle path is on a plane that is oblique to the acquired axial images, the target moves with respiration, the procedure is a lung biopsy, the target is difficult to reach (due to small size or depth of location) or the needle path is very narrow. Difficult interventions are associated with an increased risk of major complications and of procedure failure and also with an increase in the number of control scans required compared with standard interventions. Because these risks have a direct impact on the three criteria that constitute the primary outcome of the study, respectively, safety, efficacy and performance, the random assignment of the patient into the experimental or comparator arm of the trial is stratified by the expected difficulty of the procedure as well as by centre.

The expected difficulty of the procedure is entered into the electronic case report forms (eCRFs) provided by Medsharing (Fontenay Sous Bois, France; www.medsharing.fr), and the patient is then randomly assigned to the experimental (NAV) or comparator (CT) arm of the trial. The randomisation lists are generated by Medsharing using a randomisation by minimisation algorithm. By acquiring the CT scan before randomisation, it can be verified that the originally prescribed procedure is maintained and that the navigation system is available and working before the patient is assigned to the CT or NAV group, thereby minimising the later imputation of missing data for the statistical analysis.

Once the patient has been assigned to the CT or NAV group, the procedure prescribed can be carried out. For patients assigned to the CT group (procedures carried out under conventional CT guidance), the ideal needle path trajectory is defined on the acquired CT scan using the CT console. The needle entry point is identified and marked on the patient’s skin, and the sterile area is set up. A local anaesthetic is administered, and the radiologist carries out the procedure, iteratively verifying the correct position of the needle by acquiring control scans until the target has been reached. A final control scan is acquired that shows the position of the needle at the target before the procedure is continued to carry out the prescribed gesture (e.g., biopsy, abscess drainage). Any further control scans acquired after the ‘final control scan’ are not counted for this study.

For patients assigned to the NAV group (procedures carried out using navigation assistance supplied by IMACTIS-CT®), first the navigation system is installed beside the CT scanner and connected to the network. The system can then be started up, followed by the software. The electromagnetic receiver is attached to the non-sterile needle holder, and the images of the acquired CT scan are automatically transferred to the navigation system via the network. Automatically, the arrival of the images is detected, the coherence of the series is verified and the registration of the images is carried out. The navigation system enables the radiologist to define the ideal needle path trajectory directly on the patient by using the needle holder to navigate within the acquired CT volume. Once the ideal trajectory has been defined, the entry point is marked on the patient’s skin, and the sterile area is set up. The navigation system is then made sterile by removing the electromagnetic receiver from the non-sterile needle holder, placing the receiver in a sterile cover, and attaching it to the sterile needle holder. A local anaesthetic is then administered, and the radiologist carries out the procedure using navigation assistance until the target has been reached. Control scans can be acquired at any moment. A final control scan is acquired that shows the position of the needle at the target before the procedure is continued.

**Fig. 2:**
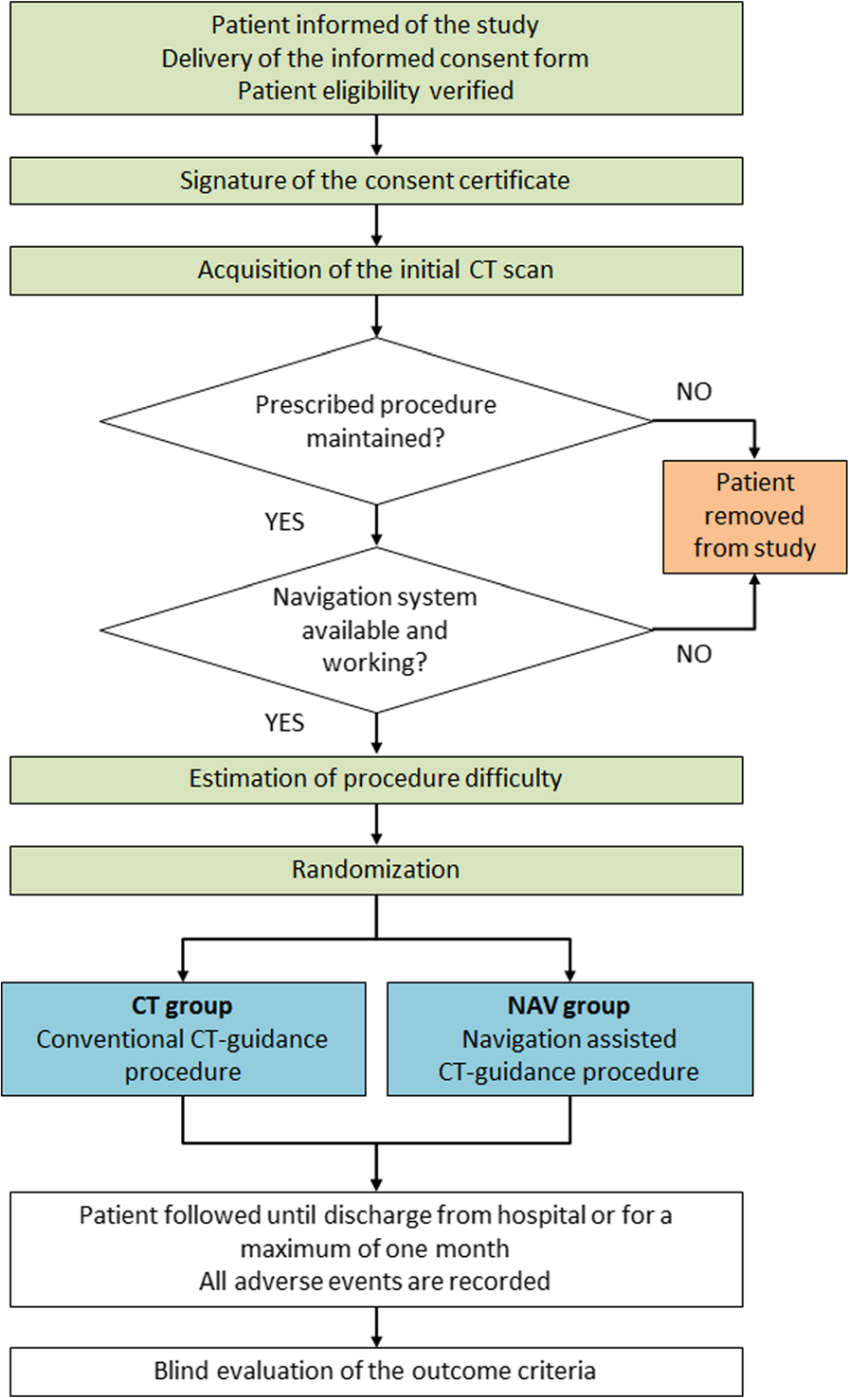
Clinical trial overview. CT Computed tomography, *CT group* Active comparator arm in which procedures are carried out guided by conventional computed tomography, *NAV group* Experimental arm of the trial in which procedures are carried out using navigation assistance

**Fig. 3:**
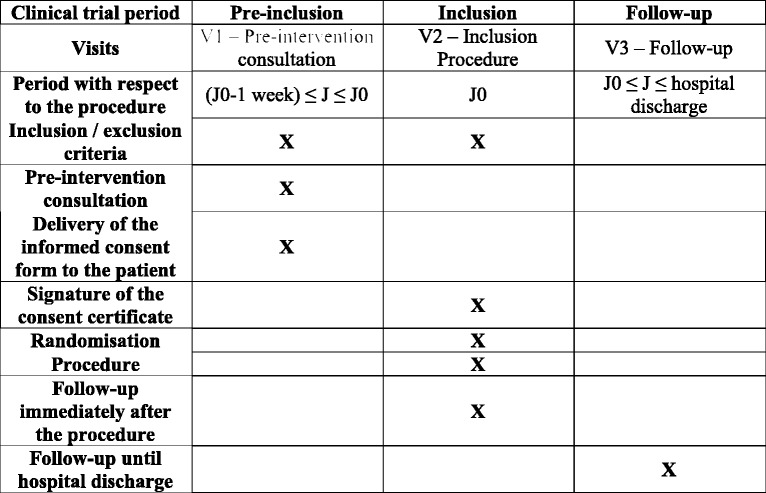
Participant timeline

Following the procedure, patients are followed until their discharge from hospital or for a maximum of 1 month. All adverse events (AEs), serious or non-serious, that occur during or after (from immediately after the procedure until hospital discharge) the procedure are recorded in the eCRFs and are declared to Grenoble-Alpes University Hospital (trial sponsor), regardless of whether the AE is attributable to the radiologist’s intervention. The AE is considered attributable if the event is directly caused by the needle insertion phase of the procedure. The AEs are classified according to the scale defined by the Society of Interventional Radiology (SIR) [30]. The study workflow and the participant timeline are presented in Figs. 2 and 3, respectively. (*See* Additional file 1 for information regarding the Standard Protocol Items: Recommendations for Interventional Trials [SPIRIT] checklist).

For every procedure, a clinical research assistant (CRA) is present with responsibility for recording the patient data in the eCRFs and later integrating the relevant medical reports (e.g., consultation, radiology, anatomopathology, hospital discharge summary reports) after they have been anonymised. All data recorded for a patient are validated by the radiologist who carried out the procedure. To minimise errors, the eCRFs were designed using range checks (to verify that entered values are within defined boundaries) and conditional logic (request/prevent entry of a field if the preceding answer is yes/no). Furthermore, during the period of patient enrolment, a data manager will verify the coherence of the data recorded and request that corrections be made when necessary. All data are monitored by Grenoble-Alpes University Hospital (trial sponsor) in order to control the occurrence of AEs and to verify that for every patient enrolled there is a signed consent certificate and that the inclusion and exclusion criteria were respected. In addition, the data included in the eCRF of every enrolled patient will be verified exhaustively. All recorded data are anonymous; only the principal investigator of each centre has access to the list of participants enrolled in his/her centre.

The trial is evaluated according to the previously stated primary and secondary outcomes whose aim is to evaluate the clinical benefit of the navigation system. The primary outcome, a combined criterion composed of three criteria to assess the safety, efficacy and performance of navigation-assisted procedures, is evaluated as follows:

- *Safety*: The number of AEs that are considered to be major (i.e., classified as C, D, E or F according to the scale defined by the SIR) and are attributable to the needle insertion phase of the procedure
- *Efficacy*: The number of targets reached; the target is considered to have been reached when the needle is positioned accurately enough to allow the next step of the procedure to be carried out
- *Performance*: The number of control scans acquired during the needle insertion phase of the procedure; that is, the number of scans acquired between and including H1 (time of the first CT scan on which the needle is visible) and H2 (time at which the needle has reached the target)

The secondary outcomes are used to assess the time required to reach the target (H2 − H1), the satisfaction of the operator with the procedure (quantitative scale) and the radiation dose delivered during the needle insertion phase of the procedure (radiation delivered between and including H1 and H2). Furthermore, because it is expected that the navigation system will provide a greater clinical benefit for difficult procedures compared with standard procedures, a subgroup analysis according to the expected difficulty of the procedure will be carried out. The usability of the device is also evaluated by analysis of the needle holder localisation files generated during the procedures.

To minimise ascertainment bias, the CT scans are post-processed before analysis such that it cannot be determined whether the scans were acquired in the experimental or comparator arm. For scans acquired with navigation assistance, the needle holder, the portion of the needle outside the body and the transmitter are erased. For scans acquired under conventional CT guidance, the portion of the needle outside the body is erased.

For each enrolled patient, the primary outcome is determined by a blinded evaluation of the post-processed CT scans and the patient’s medical reports (procedure report and hospital discharge summary), as previously described. The evaluation is carried out by a trained radiologist blinded to the patient group and to all evaluations made by the investigating radiologist who carried out the procedure. In case of discordance between the data entered by the evaluating radiologist and the investigating radiologist, the discordance is presented to an expert committee by the CRA responsible for centre and data management. The expert committee is composed of two senior radiologists who are independent from the evaluating and investigating radiologists and who will provide a consensus in case of discordance.

To ensure the correct progress of the trial and to verify that the proposed statistical analysis framework is appropriate, an interim analysis of the primary outcome will be performed once the first four participating hospitals have finished patient enrolment. To maintain a global cut-off of 5% for the final analysis, the interim analysis will be carried out using a cut-off of 0.1%. The results of the interim analysis will be considered by the TSC to judge whether the statistical analysis framework requires modifying. When patient enrolment is finished, the final monitoring is completed and the coherence of the recorded data is verified, the database will be frozen, and the final statistical analysis of the data will be carried out.

### Statistical analysis

The number of patients to be enrolled in the study is calculated as the maximum of the values calculated for each of the three different criteria (safety, efficacy and performance) that constitute the primary outcome, with the alpha level set to 5%. The calculations are performed as follows for each of the three criteria:

- *Safety*: The suggested threshold for all major complications resulting from percutaneous drainage or biopsy procedures is 10% [31, 32]. The threshold is increased to 20% for lung procedures [33]. Because one of the participating hospitals is a referral centre for lung procedures, it has been decided to define the threshold for all major complications as 12% for this study (calculated as the average threshold across the centres, 12 = (10 × 8 + 20 × 1)/9, rounded up to the nearest whole number). Considering an initial complication rate of 5% in each of the groups, a sample size of 384 patients (192 in each of the experimental and comparator arms of the trial) will be sufficient to demonstrate non-inferiority between the groups, respecting a non-inferiority cut-off δ = 7%, with 90% power and a 5% significance level.
- *Efficacy*: The minimum threshold for intervention success is 80%. Considering an initial success rate of 91% in each of the groups, a sample size of 380 patients (190 per group) will be sufficient to demonstrate non-inferiority between the groups in terms of success, respecting a non-inferiority cut-off δ = −10%, with 90% power and a 5% significance level.
- *Performance*: Considering that use of the navigation system will decrease the number of control scans acquired by 20% compared with conventional procedures (estimated from previous clinical trial), a sample size of 222 patients (111 per group) will be sufficient to demonstrate superiority in the NAV group compared with the CT group in terms of a decrease in the number of control scans acquired, with 90% power and a 5% significance level.

Estimating the loss to follow-up and the withdrawal of consent to be 4%, a minimum of 200 participants will be enrolled per group. Because the study was initially designed with the participation of eight hospitals, and to minimise a centre bias, the number of patients to be included per hospital was set at 50 ± 15. After inclusion had begun, a ninth centre requested to participate in the study. Because inclusion had already begun, the number of inclusions per centre was maintained, and the maximum sample size was defined to be 500. The study protocol was therefore modified, and the modifications were approved by the French Health Authority (Agence Nationale de Sécurité des Médicaments et des Produits de Santé) and the relevant ethics committee (Comité de Protection des Personnes, Sud-Est V, France). The sample size calculations were carried out using nQuery Advisor® 7.0 software (Statistical Solutions, Cork, Ireland) with the method ‘upper confidence limit for difference in proportions (simulation)’ [34] for the safety and efficacy criteria and Noether’s method ‘sample size determination for some common nonparametric statistics’ [35] for the performance criterion.

For the statistical analysis of the data, because non-inferiority tests are used to evaluate the safety and efficacy criteria, a PP analysis will be carried out on the data, comparing the 90% two-sided confidence interval of the difference between the proportions with the non-inferiority cut-off. Conversely, because a superiority test is used to evaluate the performance criterion, an ITT analysis will be carried out on this data, comparing the means of the two groups using Student’s *t* test or the non-parametric Mann-Whitney *U* test. For all calculations, a threshold of *p* < 0.05 is considered significant.

For the secondary outcome analysis, the means of the two groups will be compared using Student’s *t* test or the non-parametric Mann-Whitney *U* test for each outcome. Furthermore, subgroup analyses according to the difficulty of the procedure will be carried out. ITT and PP analyses will be carried out for the secondary outcomes to ensure the robustness of the results [36].

If a patient withdraws consent before random assignment to a group, that patient is removed from the study and is excluded from the statistical analysis. If other situations occur after random assignment to a group (consent is withdrawn, the protocol is not respected, the radiologist chooses to use conventional CT guidance rather than the navigation system for a patient in the NAV group, or the radiologist changes the original procedure prescribed), the patient concerned will be excluded from PP analyses. For the ITT analyses, missing data in the NAV group will be filled using the worst values for procedures of the same difficulty rating in the same centre in the NAV group, whereas missing data in the CT group will be filled using the best values for procedures of the same difficulty rating in the same centre in the CT group. If after random assignment to the NAV group the navigation system is unavailable, the patient remains in the study, and the procedure is performed under conventional CT guidance; the patient will be excluded from PP analyses. For the ITT analyses, the patient remains in the NAV group, with missing data filled using the worst values for procedures of the same difficulty rating in the same centre in the NAV group.

## Discussion

Navigation assistance systems have demonstrated their value in the fields of orthopaedics, urology and neurosurgery. A previous clinical trial demonstrated that use of the IMACTIS-CT® navigation system during CT-guided interventional procedures improved needle placement accuracy significantly and reduced the number of control CT scans acquired. The aim of the present trial is to address the lack (to the best of our knowledge) of published high-level evidence studies involving evaluation of navigation-assisted, CT-guided interventional procedures. The trial is designed to evaluate the clinical benefit, in terms of safety, efficacy and performance, of navigation-assisted, CT-guided procedures. It is expected that the more accurate needle placement achieved using the navigation system will decrease the procedure duration and decrease the radiation exposure delivered. This trial is important because it addresses the problems associated with conventional CT guidance—inaccurate needle placement, radiation exposure—and is particularly relevant because the number of interventional radiological procedures carried out in routine clinical practice is increasing.

The trial does present certain limitations, however. Firstly, the operators are not stratified according to experience, reducing the comparability of the results in the two groups. However, because the patients are randomly assigned to the experimental or comparator arm, and considering the small number (two to five) of investigators participating in the study per centre, it is expected that the distribution of experienced operators is comparable in both groups. Furthermore, training is provided for the participating radiologists and is obligatory before authorisation is granted to carry out a navigated procedure on a patient enrolled in the trial. Another limitation is that the assignment to the experimental or comparator arm is open label for the radiologist. The assignment cannot be made double-blind, owing to the nature of the procedure. However, the criteria of the primary outcome will be evaluated by a blinded expert committee after the CT scans have been post-processed, such that it cannot be determined whether the scans were acquired in the experimental or comparator arm. A third limitation concerns the absence of respiratory motion tracking integrated into the navigation system; a breath-hold approach is therefore required for certain procedures (e.g., lung procedures). An interesting future perspective is therefore the development and inclusion of respiratory motion management into the system, which is expected to improve the system’s accuracy. Finally, because the navigation system was designed to assist CT-guided interventional procedures, the system has no multi-modal capability.

## Trial status

Patient enrolment started in December 2013 and was expected to be completed before the end of June 2017.

## Additional file

Additional file 1: SPIRIT 2013 checklist: recommended items to address in a clinical trial protocol and related documents. (DOC 122 kb)

## Abbreviations

AE: Adverse event
CE: Conformité Européenne (European Conformity)
CIC-IT: Clinical Investigation Centre for Innovative Technology
CRA: Clinical research assistant
CT: Computed tomography
CT group: Active comparator arm in which procedures are carried out guided by conventional computed tomography
eCRF: Electronic case report form
ITT: Intention to treat
MRI: Magnetic resonance imaging
NAV group: Experimental arm of the trial in which procedures are carried out using navigation assistance
PP: Per protocol
SIR: Society of Interventional Radiology
SPIRIT: Standard Protocol Items: Recommendations for Interventional Trials
TIMC-IMAG: Techniques de l’Ingénierie Médicale et de la Complexité – Informatique, Mathématiques et Applications, Grenoble (Medical Engineering and Complexity Techniques – Computer Science, Mathematics and Applications, Grenoble)
TSC: Trial steering committee
US: Ultrasound
